# Left ventricular hypertrabeculation/noncompaction with epilepsy, other heart defects, minor facial anomalies and new copy number variants

**DOI:** 10.1186/1471-2350-13-60

**Published:** 2012-07-25

**Authors:** Bert Nagel, Ursula Gruber-Sedlmayr, Sabine Uhrig, Claudia Stöllberger, Eva Klopocki, Josef Finsterer

**Affiliations:** 1 Department of Pediatric Cardiology, Medical University Graz, University Children’s Hospital, Auenbruggerplatz 30, A-8036 Graz, Austria; 2 Department of Pediatrics (U. Gruber-Sedlmayr), Medical University Graz, Auenbruggerplatz 30, A-8036 Graz, Austria; 3 Institute of Human Genetics, Medical University of Graz, Harrachgasse 21/8, A-8010 Graz, Austria; 4 Krankenanstalt Rudolfstiftung, Juchgasse 25, A-1030 Wien, Austria; 5 Institute for Medical Genetics and Human Genetics, Charité Universitätsmedizin Berlin, Augustenburger Platz 1, D-13353 Berlin, Germany

**Keywords:** Cardiomyopathy, Congenital heart disease, Neurology, Pediatrics, Array CGH, Hypertrabeculation, Seizures

## Abstract

**Background:**

Left ventricular hypertrabeculation/noncompaction (LVHT) is a cardiac abnormality of unknown etiology which has been described in children as well as in adults with and without chromosomal aberrations. LVHT has been reported in association with various cardiac and extracardiac abnormalities like epilepsy and facial dysmorphism.

**Case presentation:**

A unique combination of LVHT, atrial septal defect, pulmonary valve stenosis, aortic stenosis, epilepsy and minor facial anomalies is presented in a 5.5 years old girl. Microarray-based genomic hybridization (array-CGH) detected six previously not described copy number variants (CNVs) inherited from a clinically unaffected father and minimally affected mother, thus, most likely, not clinically significant but rare benign variants.

**Conclusions:**

Despite this complex phenotype de novo microdeletions or microduplications were not detected by array CGH. Further investigations, such as whole exome sequencing, could reveal point mutations and small indels as the possible cause.

## Background

Left ventricular hypertrabeculation/noncompaction (LVHT) is a cardiac abnormality of unknown etiology which has been described in children as well as in adults. LVHT has been reported in association with various cardiac abnormalities, like Ebstein anomaly, pulmonary stenosis or atrial septal defect [1-12]. LVHT is associated with several extracardiac, especially neurological, abnormalities. LVHT has been reported in association with epilepsy, facial dysmorphism or minor anomalies [1,4,12-18]. Several chromosomal aberrations have been identified in LVHT associated with heart defects [1,4,8,10,12,15-17]. We present a pediatric patient with LVHT and other right and left heart defects, epilepsy and minor facial anomalies in whom microarray-based comparative genomic hybridization (array CGH) detected new copy number variants (CNVs).

## Case presentation

A 5.5-year old girl had undergone surgical patch closure of a large secundum atrial septal defect with valvotomy of a moderate pulmonary stenosis at the age of 2 months; a mild aortic stenosis with a thickened tricuspid aortic valve was not corrected. She was born as the first child to non-consanguineous parents. The family history was negative for sudden cardiac death, but the maternal grandfather had epileptic seizures without fever in his youth. At the age of 12 months the girl suffered from a first afebrile seizure and at the age of 23 months from a first febrile seizure. Four months later she suffered from a series of afebrile seizures. Therefore valproic acid was started and maintained for 2 years. During this treatment she was seizure-free with normal electroencephalogram (EEG). After tapering valproic acid over a period of 2 months, short generalized paroxysmal discharges appeared on the EEG for the first time without clinical correlate. One year later she suffered from two further uncomplicated febrile seizures. Since then she is seizure-free without anticonvulsive treatment but the 24 h-EEG persistently shows subclinical absences. Since the intellectual and physical development of the patient is normal it was decided together with the parents not to restart the antiepileptic medication but to follow her up closely. Cerebral magnetic resonance imaging was normal. Clinical cardiologic, echocardiographic and neurologic examinations of the first degree relatives were normal except for the mother who showed epicanthic folds and hypertelorism. Unfortunately, the maternal grandfather did not consent with any investigations.

**Figure 1:**
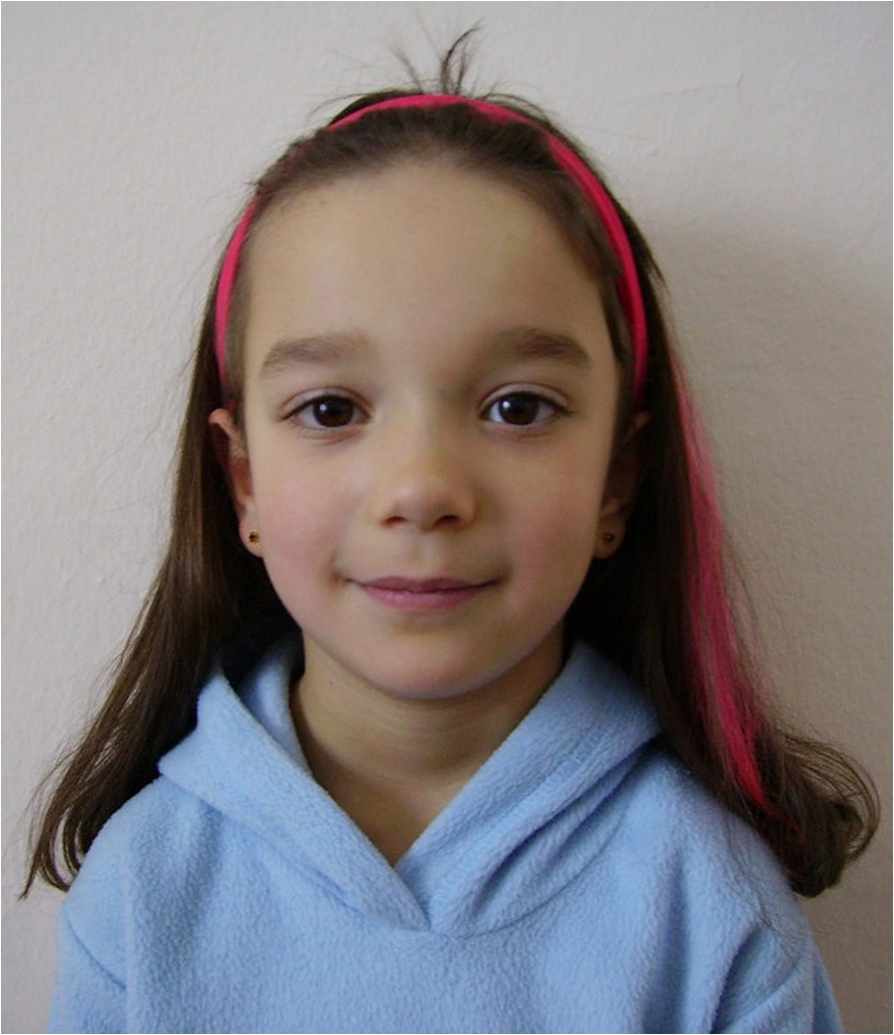
Frontal pictures of the patient showing bilateral epicanthic folds, broad eyebrows, broad nasal tip and hypertelorism.

**Figure 2:**
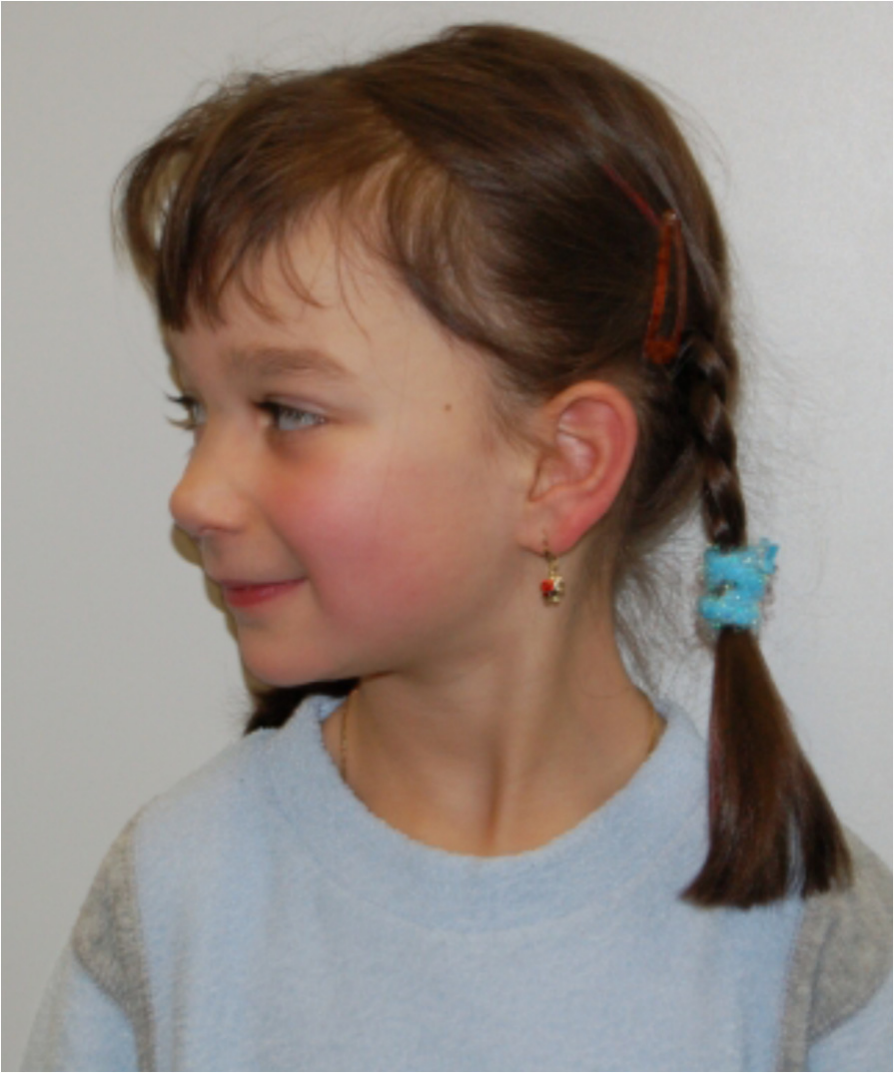
Lateral pictures of the patient showing bilateral epicanthic folds, broad eyebrows, broad nasal tip and hypertelorism.

**Figure 3:**
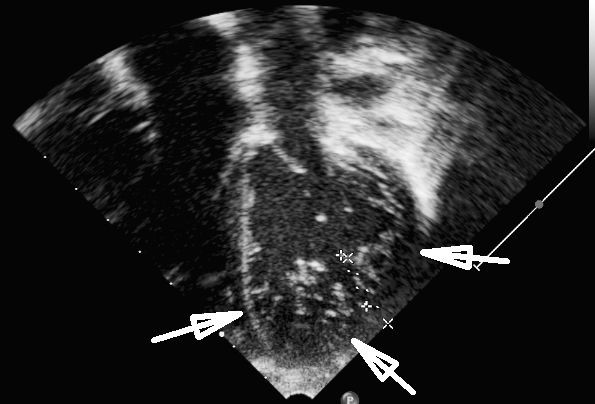
Echocardiographic apical 4-chamber-view showing the hypertrabeculated left ventricular apex with a two-layered structure.

**Figure 4:**
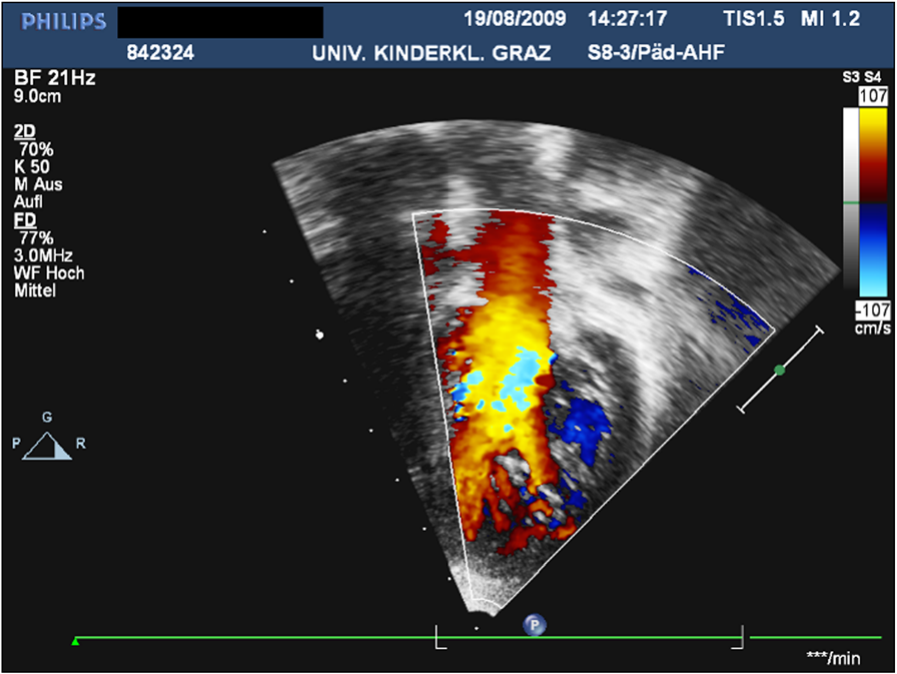
Using colour-Doppler-sonography the intertrabecular recesses are perfused from the ventricular cavity.

At inspection, she had bilateral epicanthic folds; broad eyebrows, a broad nasal tip and hypertelorism, however no ptosis or low-set ears (Figures 1 and 2). Her body weight was 18 kg (25^th^ percentile), head circumference 51 cm (50^th^ percentile) and height 115 cm (75^th^ percentile). She did not complain about any cardiac symptoms and did not suffer from heart failure. Twelve-lead electrocardiogram showed normal sinus rhythm alternating with an atrio-ventricular-nodal rhythm and an incomplete right-bundle-branch-block. Twenty-four-hour electrocardiogram showed sporadic ventricular and supraventricular ectopic beats. Echocardiography revealed a mild residual valvular pulmonary stenosis with moderate pulmonary valve regurgitation and mild aortic valve stenosis with mild aortic regurgitation. Left ventricular function was normal, however, extensive hypertrabeculation resulting in a two-layered structure of the myocardium was visible in the mid-ventricular and apical segments of the left ventricle, consistent with the diagnosis of LVHT (Figures 3 and 4). Review of previous echocardiographic examinations disclosed that LVHT had been present already at age 2 years. As a primary prophylaxis for cardiac embolism aspirin 50 mg/d was started. Since she was symptom-free, no further cardiac medication was prescribed. At the latest follow-up investigation in September 2011, she was in a good cardiac and neurologic condition, no seizures had recurred and her intellectual development was normal. Echocardiography was unchanged.

Molecular genetic analyses of the *PTPN11, KRAS, RAF1 and SOS1* genes were negative. Array CGH using a 1 M oligonucleotide microarray platform (Agilent Technologies, Santa Clara, CA) was performed to screen genome-wide for submicroscopic deletions and duplications [19]. Genomic positions are given according to genome-build hg18. Array CGH detected five previously not described CNVs as listed in Table 1. FISH analyses were not carried out because of the size of the CNVs. Regarding the duplications it could not be assessed whether they were tandem-duplications on the same locus or whether the duplicated fragment was inserted or translocated in another chromosome. By application of quantitative Real-Time PCR (qPCR) we could show, however, that none of the changes had developed de novo. All five CNVs are currently not listed in the Toronto Database of Genomic Variants (DGV), therefore, have to be considered as non-frequent variants in normal controls. Follow-up and testing of the index patient and her parents by qPCR revealed that all CNVs were inherited from one of the unaffected parents (maternal: deletion 1q42.3, duplication 3q26.32q26.33; paternal: duplication 14q32.11, deletion and duplication 20q13.33). We thus conclude that these changes are most likely not clinically significant CNVs but rare benign variants, although a reduced penetrance of inherited CNVs cannot be excluded.

**Table 1:** Array CGH detected CNVs

**Chromosomal band**	**CNV**	**Start**	**End**	**Genes**	
1p12	del	120338195	120404356	*NOTCH2* (intragenic, exon 2–4)	homolog of Drosophila Notch2, MIM 600275
1q42.3	del	234409418	234422919	*GPR137B* (intragenic, exon 5)	G protein-coupled receptor 137B, MIM 604658
3q26.32q26.33	dup	180445165	180668097	*KCNMB3* (5' partial), *ZNF639*, *MFN1*, *GNB4*	potassium large conductance calcium-activated channel, subfamily M beta member 3, MIM 605222; zinc finger protein 639; Mitofusin 1, MIM 608506; Guanine nucleotide-binding protein beta-4, MIM 610863
14q32.11	dup	89942827	90019073	*CALM1* (3' partial)	Calmodulin 1, MIM 114180
20q13.33	del	59412001	59460188	*CDH4* (intronic)	cadherin 4 (retinal cadherin), MIM 603006
20q13.33	dup	59597204	59629701	*CDH4* (intronic)	

Positions according to hg18.

## Discussion

LVHT associated with atrial septal defect with or without pulmonary valve stenosis has been reported by several authors [2-9,11]. In most of these patients atrial septal defect and LVHT were associated with several other cardiac and extracardiac anomalies, and genetic studies revealed various mutations as listed in Table 2. However, the combination of a secundum type atrial septal defect with pulmonary stenosis and aortic stenosis in combination with LVHT has not been described to date. LVHT associated with seizures has been reported in children with monosomy 1p36 [1,15,16] and interstitial 1q43 deletion [4]. Facial anomalies and LVHT have been described previously in children with developmental impairment, interstitial 1q43 deletion [4], deletion 1p36 syndrome [1], interstitial 8p23.1 deletion [12], point mutations and deletions of the *NSD1* gene located at chromosome 5q35 [17] and in an adult with sex chromosome mosaicism, male phenotype and the karyotype mos45,X(28)/46,X,+mar(21)/47,X, + 2 mar(1) [18].

**Table 2:** Reports about left ventricular noncompaction/hypertrabeculation with atrial septal defect

**Ref.**	**Age/sex**	**Additional cardiac findings**	**Extracardiac comorbidity**	**Follow-up**	**Genetic findings**
[2]	39y/f	Eb, heart failure, EF <55	NI	NI	*MYH7* mutation
[9]	16y/m	Right pulmonary vein aplasia, left-sided pulmonary vein obstruction	Right lung hypoplasia	Balloon dilatation, interventional ASD closure	NI
[10]	NI/m	AV-block I, syncope	NI	NI	*NKX2.5* mutation
[11]	23y/m	VSD	NI	NI	NI
[3]	Birth/f	VSD	Agenesis of corpus callosum, facial dysmorphism, febrile seizures	Alive at 3 years, delayed psychomotor development	Interstitial 1q43 deletion
[5]	16y/m	None	NI	NI	E101K *ACTC* mutation
[5]	62y/m	None	NI	NI	E101K *ACTC* mutation
[6]	1 m/m	None	Hypotonia, developmental delay, nystagmus, strabismus, failure to thrive	NI	*MMACHC* mutation
[7]	5d/m	EF <55, PDA, Eb	NI	Alive	NI
[7]	1d/f	None	Ni	Alive	NI
[7]	6 m/m	Ectopic atrial rhythm, VSD	NI	Alive	NI
[7]	5y/f	WPW, Eb	NI	Alive	NI
[7]	6 m/f	EF <55, VSD, SAS, Coa	NI	Dead	NI
[7]	2 m/m	VSD	NI	Alive	NI
[7]	1 m/f	SVT, VT, VSD, PDA, DILV	NI	Alive	NI
[7]	Birth/f	EF <55, PDA	NI	Dead	NI
[7]	1d/f	VSD, PDA, BAV, LSVC	NI	Alive	NI
[7]	9 m/f	EF <55, VSD	NI	Dead	NI
[7]	2d/m	EF <55, VSD	NI	Dead	NI
[7]	3 m/m	VSD	NI	Alive	NI
[7]	6d/m	EF <55	NI	Alive	NI
[7]	9 m/m	VSD	NI	Alive	NI
[7]	7d/f	EF <55	NI	Alive	NI
[7]	1 m/m	EF <55	NI	Alive	NI
[8]	Birth/f	Sinusbradycardia, pulmonary valve atresia	BLI, ACV, abdominal situs ambiguous, polysplenia	Dead	Linkage to 6p24.3-21.2
[8]	22y/m	Sinusbradycardia, AF	BLI, ACV	Alive	Linkage to 6p24.3-21.2
[8]	32y/f	Pulmonary valve stenosis, sick sinus syndrome	ACV, polysplenia, malrotation of the gut	Alive	Linkage to 6p24.3-21.2
[8]	59y/m	AF, heart failure, EF <55	Polysplenia	Died suddenly	Linkage to 6p24.3-21.2

NI, not indicated; EF, <55 left ventricular ejection fraction <55 %; PDA, patent ductus arteriosus; Eb, Ebstein anomaly of the tricuspid valve; VSD, ventricular septal defect; WPW, Wolff-Parkinson-White; SAS, subaortic stenosis; Coa, coarctation of the aorta; SVT, supraventricular tachycardia; VT, ventricular tachycardia; DILV, double-inlet left ventricle; BAV, bicuspid aortic valve; LSVC, left-sided superior vena cava; ACV, azygous continuation of the vena cava inferior; AF, atrial fibrillation; BLI, bronchial left isomerism.

CNVs are structural genomic variants due to deletions or duplications, resulting in a copy-number change of the respective genomic region. CNVs may include entire genes, regions of transcribed sequence, or nontranscribed sequences. Whereas the duplication or deletion of a gene can be expected to have an effect on gene dosage, the consequences of CNVs in nontranscribed sequences are less clear [20]. According to the resolution of the applied array platform (5–10 kb genome-wide) we currently do not have evidence for a clinically relevant chromosomal imbalance i.e. gain or loss of genomic material. However, it is still possible that an underlying chromosomal abnormality either below the detection limit of the array or a balanced rearrangement which is undetectable by array CGH is causative for the described phenotype. This assumption is supported by the previously reported chromosomal defects in LVHT associated with facial anomalies, atrial septal defect and epilepsy. The presence of a chromosomal abnormality as the underlying defect is further substantiated by the fact that LVHT is particularly prevalent in young patients with chromosomal abnormalities [21].

In patients with epilepsy, an association with a truncation mutation of the *KCNMB3* gene has been described which is partly affected from the duplication on chromosome 3 [22]. However, consequences of the duplication on the transcripts of this gene were not tested, and this is why such a presumed association remains speculative.

The first CNV detected in our patient (1p12 deletion) results in a deletion of part of the *NOTCH2* gene that can be mutated in Alagille syndrome, a multisystem disorder with predominantly liver-, skeletal, ophthalomologic and renal abnormalities [23]. We consider Alagille syndrome unlikely in our patient since she did not show any of these abnormalities, although highly variable expressivity of the affected systems has been described in subjects with NOTCH2 mutations [24].

## Conclusions

This case shows that childhood LVHT may be associated with other cardiac abnormalities, central nervous system disease and minor facial anomalies. Despite this complex phenotype de novo microdeletions or microduplications were not detected by array CGH. Further investigations, such as whole exome sequencing, could reveal point mutations and small indels as the possible cause.

### Consent

The father of the patient has given his consent for the case report to be published.

## Methods

### Microarray-based comparative genomic hybridization (Array CGH)

Array CGH was performed using the 1 M oligonucleotide array (SurePrint G3 Human CGH 1x 1 M microarray, Agilent Technologies, Santa Clara, CA). We used female reference DNA (Human Genomic DNA female, Promega). Processing of the array was done according to the manufacturer's instructions. Extraction of microarray TIFF images had been done by “Feature Extraction” and the following data analysis was done by the “DNA Analytics 4.0” software (both Agilent Technologies, Santa Clara, CA). The following analysis settings in DNA analytics software were applied: algorithm ADM-2, filter 5 probes, log2ratio 0.29.

### Quantitative real-time PCR (qPCR)

Genomic DNA samples of the index patient and her parents were obtained from EDTA-blood. Amplicons were located within the aberrant regions as detected by array CGH and in flanking regions. Primer sequences can be obtained upon request. qPCR was performed as previously described [20].

## Abbreviations

Array CGH: Microarray-based comparative genomic hybridization; CNVs: Copy number variants; LVHT: Left ventricular hypertrabeculation/noncompaction.

## Competing interest

The authors declare that they have no competing interests.

## Authors’ contributions

BN collected clinical data, performed follow-up investigations, drafted the manuscript. UG-S collected clinical data, performed follow-up investigations, drafted the manuscript. SU performed the description of the dysmorphic features and took the photographs. CS drafted the manuscript, performed literature research, corresponding author. EK carried out the molecular genetic studies and drafted the manuscript. JF drafted the manuscript and performed literature research. All authors read and approved the final manuscript.

## Pre-publication history

The pre-publication history for this paper can be accessed here:

http://www.biomedcentral.com/1471-2350/13/60/prepub

## References

[B1] Battaglia A Hoyme HE Dallapiccola B Zackai E Hudgins L McDonald-McGinn D Bahi-Buisson N Romano C Williams CA Brailey LL Zuberi SM Carey JC Further delineation of deletion 1p36 syndrome in 60 patients: a recognizable phenotype and common cause of developmental delay and mental retardation Pediatrics 2008 121 404 410 10.1542/peds.2007-0929 18245432

[B2] Budde BS Binner P Waldmüller S Höhne W Blankenfeldt W Hassfeld S Brömsen J Dermintzoglou A Wieczorek M May E Kirst E Selignow C Rackebrandt K Müller M Goody RS Vosberg HP Nürnberg P Scheffold T Noncompaction of the ventricular myocardium is associated with a de novo mutation in the beta-myosin heavy chain gene PLoS One 2007 2 e1362 10.1371/journal.pone.0001362 18159245 PMC2137931

[B3] Fazio G Pipitone S Iacona MA Marchi S Mongiovi M Zito R Sutera L Novo S The noncompaction of the left ventricular myocardium: our paediatric experience J Cardiovasc Med 2007 8 904 908 10.2459/JCM.0b013e32801462b0 17906475

[B4] Kanemoto N Horigome H Nakayama J Ichida F Xing Y Buonadonna AL Kanemoto K Gentile M Interstitial 1q43-q43 deletion with left ventricular noncompaction myocardium Eur J Med Genet 2006 49 247 253 10.1016/j.ejmg.2005.06.004 16762826

[B5] Monserrat L Hermida-Prieto M Fernandez X Rodríguez I Dumont C Cazón L Cuesta MG Gonzalez-Juanatey C Peteiro J Alvarez N Penas-Lado M Castro-Beiras A Mutation in the alpha-cardiac actin gene associated with apical hypertrophic cardiomyopathy, left ventricular non-compaction, and septal defects Eur Heart J 2007 28 1953 1961 10.1093/eurheartj/ehm239 17611253

[B6] Profitlich LE Kirmse B Wasserstein MP Diaz GA Srivastava S High prevalence of structural heart disease in children with cb1C-type methylmalonic aciduria and homocystinuria Mol Genet Metab 2009 98 344 348 10.1016/j.ymgme.2009.07.017 19767224

[B7] Tsai SF Ebenroth ES Hurwitz RA Cordes TM Schamberger MS Batra AS Is left ventricular noncompaction in children truly an isolated lesion? Pediatr Cardiol 2009 30 597 602 10.1007/s00246-008-9382-1 19184169

[B8] Wessels MW De Graaf BM Cohen-Overbeek TE Spitaels SE de Groot-de Laat LE Ten Cate FJ Frohn-Mulder IF de Krijger R Bartelings MM Essed N Wladimiroff JW Niermeijer MF Heutink P Oostra BA Dooijes D Bertoli-Avella AM Willems PJ A new syndrome with noncompaction cardiomyopathy, bradycardia, pulmonary stenosis, atrial septal defect and heterotaxy with suggestive linkage to chromosome 6p Hum Genet 2008 122 595 603 10.1007/s00439-007-0436-x 17938964

[B9] De Pasquale Meyer G Kretschmar O Valsangiacomo Buechel ER Kellenberger C Bauersfeld U Attenhofer Jost CH Rare combination of congenital aplasia of the right pulmonary veins, left ventricular noncompaction, partial membranous obstruction of left-sided pulmonary veins and secundum atrial septal defect Int J Cardiol 2011 152 e49 51 10.1016/j.ijcard.2010.12.002 21242010

[B10] Ouyang P Saarel E Bai Y Luo C Lv Q Xu Y Wang F Fan C Younoszai A Chen Q Tu X Wang QK A de novo mutation in NKX2.5 associated with atrial septal defects, ventricular noncompaction, syncope and sudden death Clin Chim Acta 2011 412 170 175 10.1016/j.cca.2010.09.035 20932824 PMC2998397

[B11] Sakan H Okayama S Uemura S Somekawa S Ishigami K Takeda Y Kawata H Horii M Fujimoto S Saito Y Atrial right-to-left shunt without pulmonary hypertension in a patient with biventricular non-compaction cardiomyopathy accompanied by ventricular and atrial septal defects Intern Med 2011 50 1747 1751 10.2169/internalmedicine.50.5290 21841338

[B12] Blinder JJ Martinez HR Craigen WJ Belmont J Pignatelli RH Jefferies JL Noncompaction of the left ventricular myocardium in a boy with a novel chromosome 8p23.1 deletion Am J Med Genet A 2011 155 2215 2220 10.1002/ajmg.a.34129 21834050

[B13] Hussein A Schmaltz AA Trowitzsch E Isolierte Fehlentwicklung (“Noncompaction“) des Myokards bei drei Kindern Klin Pädiatr 1999 211 175 178 10.1055/s-2008-1043782 10412129

[B14] Finsterer J Stöllberger C Gelpi E Successful heart failure therapy in mitochondrial disorder with noncompaction cardiomyopathy Int J Cardiovasc Imaging 2006 22 393 398 10.1007/s10554-005-9073-4 16502323

[B15] Saito S Kawamura R Kosho T Shimizu T Aoyama K Koike K Wada T Matsumoto N Kato M Wakui K Fukushima Y Bilateral perisylvian polymicrogyria, periventricular nodular heterotopia, and left ventricular noncompaction in a girl with 10.5–11.1 Mb terminal deletion of 1p36 Am J Med Genet A 2008 146A 2891 2897 10.1002/ajmg.a.32556 18925681

[B16] Thienpont B Mertens L Buyse G Vermeesch JR Devriendt K Left-ventricular non-compaction in a patient with monosomy 1p36 Eur J Med Genet 2007 50 233 236 10.1016/j.ejmg.2007.01.002 17337261

[B17] Martinez HR Belmont JW Craigen WJ Taylor MD Jefferies JL Left ventricular noncompaction in Sotos syndrome Am J Med Genet A 2011 155A 1115 1118 21484993 10.1002/ajmg.a.33838

[B18] Altenberger H Stöllberger C Finsterer J Isolated left ventricular hypertrabeculation /noncompaction in a Turner mosaic with male phenotype Acta Cardiol 2009 64 99 103 10.2143/AC.64.1.2034370 19317306

[B19] Klopocki E Ott CE Benatar N Ullmann R Mundlos S Lehmann K A microduplication of the long range SHH limb regulator (ZRS) is associated with triphalangeal thumb-polysyndactyly syndrome Med Genet 2008 45 370 375 10.1136/jmg.2007.055699 18178630

[B20] Klopocki E Mundlos S Copy-number variations, noncoding sequences, and human phenotypes Annu Rev Genomics Hum Genet 2011 12 53 72 10.1146/annurev-genom-082410-101404 21756107

[B21] Finsterer J Cardiogenetics, neurogenetics, and pathogenetics of left ventricular hypertrabeculation/ noncompaction Pediatr Cardiol 2009 30 659 681 10.1007/s00246-008-9359-0 19184181

[B22] Lorenz S Heils A Kasper JM Sander T Allelic association of a truncation mutation of the KCNMB3 gene with idiopathic generalized epilepsy Am J Med Genet B Neuropsychiatr Genet 2007 144B 10 13 10.1002/ajmg.b.30369 16958040

[B23] Varadkar P Kraman M Despres D Ma G Lozier J McCright B Notch2 is required for the proliferation of cardiac neural crest-derived smooth muscle cells Dev Dyn 2008 237 1144 1152 10.1002/dvdy.21502 18330927

[B24] Kamath BM Bauer RC Loomes KM Chao G Gerfen J Hutchinson A Hardikar W Hirschfield G Jara P Krantz ID Lapunzina P Leonard L Ling S Ng VL Hoang PL Piccoli DA Spinner NB NOTCH2 mutations in Alagille syndrome J Med Genet 2012 49 138 144 10.1136/jmedgenet-2011-100544 22209762 PMC3682659

